# Seed Treatment with Cold Plasma and Electromagnetic Field: Changes in Antioxidant Capacity of Seedlings in Different *Picea abies* (L.) H. Karst Half-Sib Families

**DOI:** 10.3390/plants13152021

**Published:** 2024-07-23

**Authors:** Ieva Čėsnienė, Vytautas Čėsna, Diana Miškelytė, Vitalij Novickij, Vida Mildažienė, Vaida Sirgedaitė-Šėžienė

**Affiliations:** 1 Institute of Forestry, Lithuanian Research Centre for Agriculture and Forestry, Liepų 1, LT-53101 Girionys, Lithuania; vytautas.cesna@lammc.lt (V.Č.); vaida.seziene@lammc.lt (V.S.-Š.); 2 Department of Environmental Sciences, Vytautas Magnus University, Universiteto 10, Akademija, LT-53361 Kaunas, Lithuania; diana.miskelyte@vdu.lt; 3 Institute of High Magnetic Fields, Vilnius Gediminas Technical University, Saulėtekio al. 11, LT-10223 Vilnius, Lithuania; vitalij.novickij@vgtu.lt; 4 Department of Immunology, State Research Institute Centre for Innovative Medicien, Santariskiu g. 5, LT-08406 Vilnius, Lithuania; 5 Faculty of Natural Sciences, Vytautas Magnus University, Universiteto 10, Akademija, LT-53361 Kaunas, Lithuania; vida.mildaziene@vdu.lt

**Keywords:** abiotic stress, antioxidant enzymes, antioxidant activity, biotic stress, chemistry, defense, Norway spruce, physiology

## Abstract

In the context of climate change, methods to improve the resistance of coniferous trees to biotic and abiotic stress are in great demand. The common plant response to exposure to vastly different stressors is the generation of reactive oxygen species (ROS) followed by activation of the defensive antioxidant system. We aimed to evaluate whether seed treatment with physical stressors can activate the activity of antioxidant enzymes and radical scavenging activity in young *Picea abies* (L.) H. Karst seedlings. For this, we applied seed treatment with cold plasma (CP) and electromagnetic field (EMF) and compared the response in ten different half-sib families of Norway spruce. The impact of the treatments with CP (1 min—CP1; 2 min—CP2) and EMF (2 min) on one-year-old and two-year-old *P. abies* seedlings was determined by the emergence rate, parameters of growth, and spectrophotometric assessment of antioxidant capacity (enzyme activity; DPPH and ABTS scavenging) in needles. The results indicated that the impact of seed treatment is strongly dependent on the genetic family. In the 577 half-sib family, the activity of antioxidant enzymes catalase (CAT), ascorbate peroxidase (APX), peroxidase (POX), and glutathione reductase (GR) increased after EMF-treatment in one-year-old seedlings, while similar effects in 477 half-sib family were induced by CP2 treatment. In two-year-old seedlings, CP1-treatment increased CAT, APX, POX, GR, SOD, DPPH, and ABTS activity in the 457 half-sib family. However, no significant impact of the treatment with CP1 was determined in one-year-old seedlings in this family. The application of novel technologies and the consideration of the combinatory impact of genetic and physical factors could have the potential to improve the accumulation of compounds that play an essential role in the defense mechanisms of *P. abies*. Nevertheless, for different resistance and responses to stressors of plants, their genetic properties play an essential role. A comprehensive analysis of interactions among the stress factors (CP and EMF), genetic properties, and changes induced in the antioxidant system can be of importance both for the practical application of seed treatment in forestry and for understanding fundamental adaptation mechanisms in conifers.

## 1. Introduction

Conifers are a highly resilient life form and one of the most successful organisms thanks to their evolutionary adaptations [1]. Even though, their long-life cycle and immobility increase their vulnerability to repeated abiotic and biotic stresses that affect conifer health [2,3,4]. Recent studies have examined the effects of biological control agents that can be used to control the expansion of conifer pests and pathogens or to increase stress resistance [5,6,7,8,9,10]. To increase the biologically active compounds in plant tissue, seed priming with physical stressors can be applied [11,12,13].

The application of low-temperature plasma (CP) and electromagnetic field (EMF) has a high potential in the forestry field, without a negative impact on the environment [14,15,16,17,18]. The advantage of plasma, in contrast to other stressors, is its capacity to generate active energy-containing species [13,19]. CP is comprised of charged particles, such as free electrons and ions, as well as neutral activated species, including gas molecules, free radicals, metastable particles, and produced UV photons [13,20]. EMF is a non-ionizing radiation, characterized by energy and momentum conveyed through alternating magnetic and electric fields [11]. It was recently demonstrated that seed priming with CP and EMF, besides positive effects on germination and seedling growth [13,21,22,23,24,25,26,27], also can enhance the synthesis of biologically active compounds and photosynthetic pigments in coniferous and deciduous tree species [13,18,21]. However, there is still a knowledge gap in the effects of seed treatments with CP and EMF on the antioxidant capacity in tissues of trees.

ROS are generated during metabolic processes serving essential signaling functions in living organisms [28,29,30,31]. In plants, the primary sources of ROS are the electron transport chains in chloroplasts and mitochondria, along with photorespiration in peroxisomes [31,32,33,34]. ROS impact on plants can vary depending on its concentration, the level of stress severity, the cellular energy status, and the antioxidant capacity [31,35].

The antioxidant capacity contributes to a plant’s survival when the stress levels surpass the natural defense mechanisms [36,37,38]. The plants’ ability to counteract oxidative stress relies on the effectiveness of their antioxidant system consisting of antioxidant enzymes and numerous molecular ROS scavengers like ascorbate, glutathione, etc. Superoxide dismutase (SOD; EC:1.15.1.1), catalase (CAT; EC:1.11.1.6), ascorbate peroxidase (APX; EC:1.11.1.11), peroxidase (POX; EC:1.11.1.5), and glutathione reductase (GR; EC 1.8.1.7) are the most abundant antioxidant enzymes in plants [37,39]. SOD is one of the major enzymes scavenging stress-generated free radicals, via converting superoxide •O_2_^−^ to H_2_O_2_ and O_2_ [38]. CAT is engaged in the scavenging of H_2_O_2_ produced during photorespiration [38]. APX functions both as a scavenger of H_2_O_2_ and as a sensor for redox changes within plant cells [40]. POX operates in the extracellular space to eliminate H_2_O_2_ [38]. GR is nicotinamide adenine dinucleotide phosphate (NADPH) oxidoreductase, an essential component of the ascorbate-glutathione pathway catalyzing the reduction in oxidized glutathione (GSSG) to its reduced monomeric counterpart (GSH) [38]. The assessment of the antioxidant capacity in tissues provides important information for the evaluation of the physiological capacity of the tree defense system.

On the other side, genetic inter-species differences among plants can have a significant impact on response to different stressors and plant defense. Previous studies revealed that based on different half-sib families (genotypes), coniferous trees, similar to deciduous trees, can synthesize different amounts of secondary metabolites, sugars, photosynthetic pigments, and the capacity of antioxidant system [41,42,43,44]. In conifers, the relationship between these biologically active compounds and even slight changes in their synthesis can predetermine whether the tree is under stress [18]. Therefore, the selection of *P. abies* genotypes based on the metabolic pathways in needles by comparing changes induced by seed priming with CP and EMF in the content of biologically active compounds and antioxidants in seedlings might have a potential for producing forest stands with the enhanced resistance both to biotic and abiotic stress. However, large variation between the genotypes after such stressors makes it complicated to apply procedures, and more studies are needed.

The main purpose of this study was to assess the impact of pre-sowing seed treatment with CP and EMF on the antioxidant capacity in the needles of different *P. abies* half-sib families, aiming to estimate how the antioxidant defense mechanisms are affected. We hypothesized that: (1) seed treatment with physical stressors has an impact on antioxidant capacity in one-year-old and two-year-old *P. abies* seedlings; (2) induced changes in antioxidant capacity depend on the treatment method and duration; (3) the response to the treatment is different among the studied half-sib families.

## 2. Results

### 2.1. Emergence of P. abies Seedlings

The emergence percentage of control *P. abies* seedlings varied substantially (from 36% to 88%) among half-sib families. The emergence percentage in the control and CP-treated groups was the lowest in the 599 half-sib family and the highest in the 417 half-sib family (Table 1). Large variation was also observed in CP1-, CP2- and EMF2-treated groups (from 36% to 79%, from 39% to 85%, and from 31% to 81%, respectively).

Seed treatment with CP1 did not affect seedling emergence in eight of the tested half-sib families; however, in 417 and 454 half-sib families negative effect was observed (reduced emergence by 9% and 30%, respectively). The CP2 treatment significantly increased emergence in 477 half-sib families (by 35%). However, longer treatment with CP has a negative impact on seedling emergence in 454, 541, and 577 half-sib families (reduced it by 24%, 17%, and 17%, respectively). Seed treatment with EMF resulted in a higher emergence percentage of seedlings in one family (463) compared to the control (by 21%); meanwhile, the EMF reduced emergence in the 417 and 454 half-sib families by 18% and 17%, respectively.

### 2.2. Height of P. abies Seedlings

The height of the above-ground part of the seedlings was measured to assess the growth of seedlings during both the first and second vegetation seasons. The highest height among the control one-year-old seedlings was observed in the 454 and 541 half-sib families (Table 2). Meanwhile, the highest height in the control two-year-old spruce seedlings was in the 463 half-sib family (Table 2).

CP1 effect on height of one-year-old seedlings in *P. abies* half-sib families was diverse. Four half-sib families (417, 457, 477, and 548) exhibited an increase in height by 16-19%, respectively, while seedlings from 463 families were 57% higher compared to the control (Table 3). However, two families (454 and 577) had reduced height by more than 10% due to seed treatment with the CP1. Meanwhile, a longer seed treatment (CP2) resulted in increased height in four half-sib families (457, 463, 548, and 599) by 18%, 19%, 30%, and 10%, respectively. A negative CP2 effect on height was observed in the same half-sib families as for CP1 with a reduction of 16% and 38%, respectively. EMF-treatment increased height by 13% in one half-sib family only (457) but reduced height in 417, 454, 541, and 577 half-sib families by 14%, 11%, 10%, and 33%, respectively.

Three half-sib families (417, 457, and 541) from CP1 treated groups exhibited an increase in two-years-old seedling height by 25%, 13%, and 11%, respectively. However, a longer seed treatment with CP and EMF did not have a significant positive impact on height. On the contrary, a negative effect of CP2 and EMF2 on height was observed in three half-sib families (CP2 reduced growth of 463 and 577 half-sib families; EMF2—541 half-sib family seedlings) with the height lower by more than 10%.

### 2.3. Changes of Activity of Antioxidant Enzymes in Needles of P. abies

**Figure 1:**
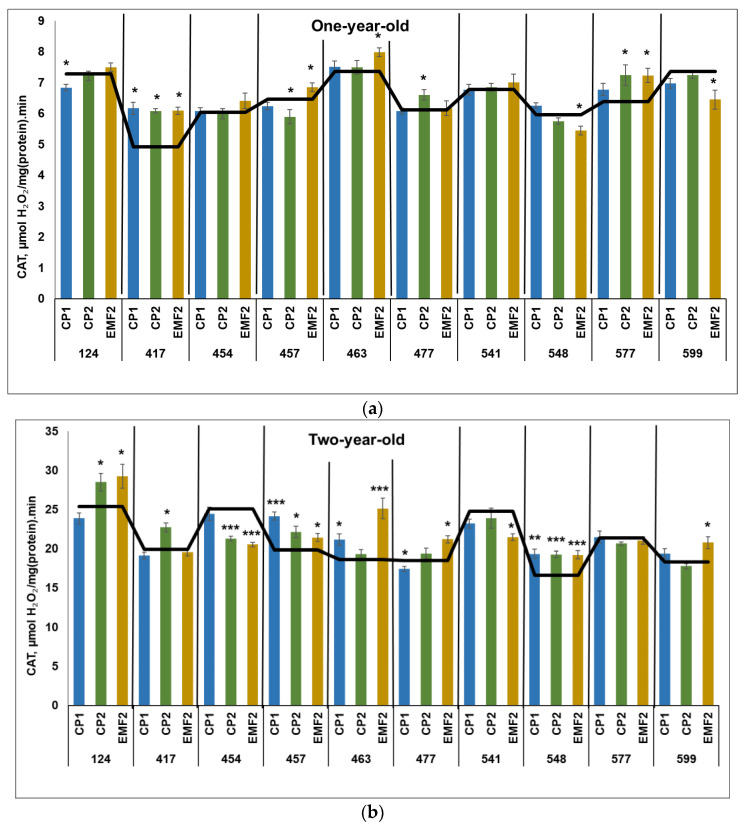
CAT activity in needles of (**a**) one-year-old and (**b**) two-year-old seedlings of different half-sib families of *Picea abies* (N = 360; 3 biological replicates × 3 technical replicates for each sample). The black line in the graphs shows the mean values of control; CP1—seed treatment with cold plasma 1 min; CP2—seed treatment with cold plasma 2 min; EMF2—seed treatment with electromagnetic field 2 min. The asterisks indicate the statistical significance of the difference (Student’s test) between the treated group (CP1, CP2, and EMF2) and the control (C) for each half-sib family (* *p* < 0.05; ** *p* < 0.01; *** *p* < 0.001).

**Figure 2:**
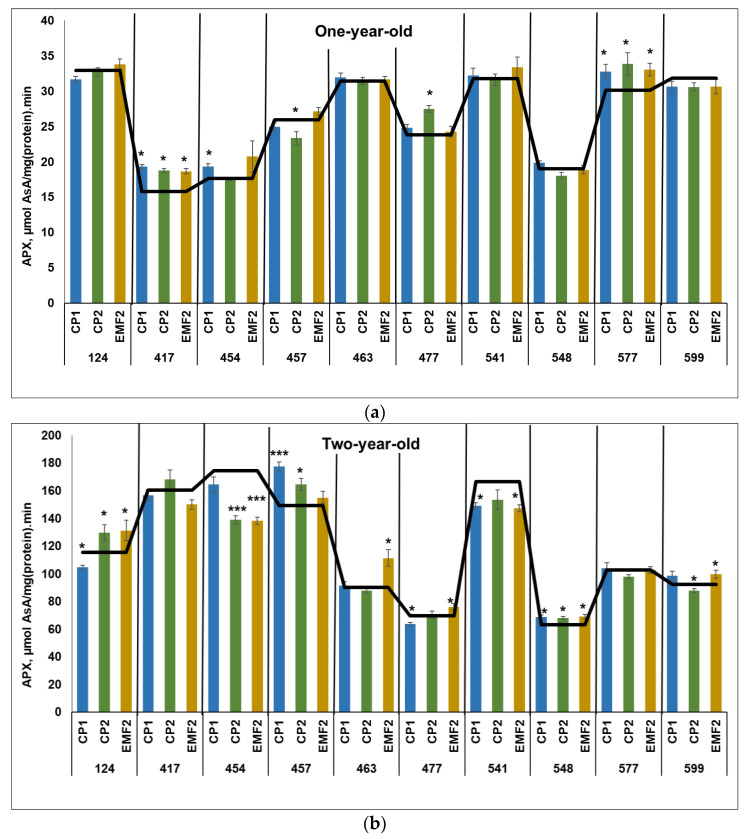
APX activity in needles of (**a**) one-year-old and (**b**) two-year-old seedlings of different half-sib families of *Picea abies* (N = 360; 3 biological replicates × 3 technical replicates for each sample). The black line in the graphs shows the mean values of control; CP1—seed treatment with cold plasma 1 min; CP2—seed treatment with cold plasma 2 min; EMF2—seed treatment with electromagnetic field 2 min. The asterisks mean the statistical significance of the difference (Student’s test) between the treated group (CP1, CP2, and EMF2) and the control (C) in each half-sib family (* *p* < 0.05; *** *p* < 0.001).

**Figure 3:**
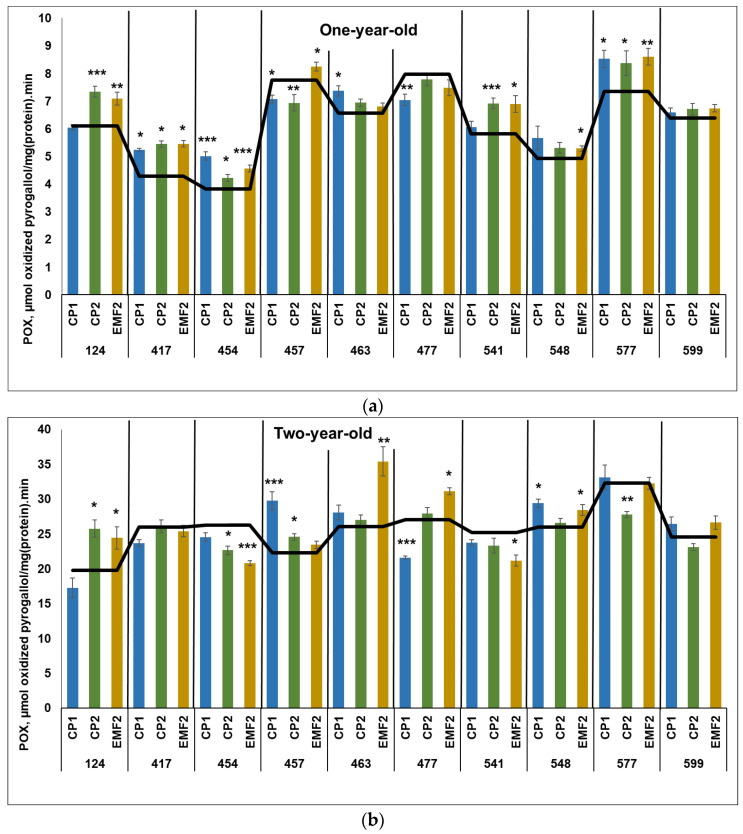
POX activity in needles of (**a**) one-year-old and (**b**) two-year-old seedlings of different half-sib families of *Picea abies* (N = 360; 3 biological replicates × 3 technical replicates for each sample). The black line in the graphs shows the mean values of control. CP1—seed treatment with cold plasma 1 min; CP2—seed treatment with cold plasma 2 min; EMF2—seed treatment with electromagnetic field 2 min. The asterisks indicate the statistical significance of the difference between (Student’s test) the treated group (CP1, CP2, and EMF2) and the control (C) in each half-sib family (* *p* < 0.05; ** *p* < 0.01; *** *p* < 0.001).

**Figure 4:**
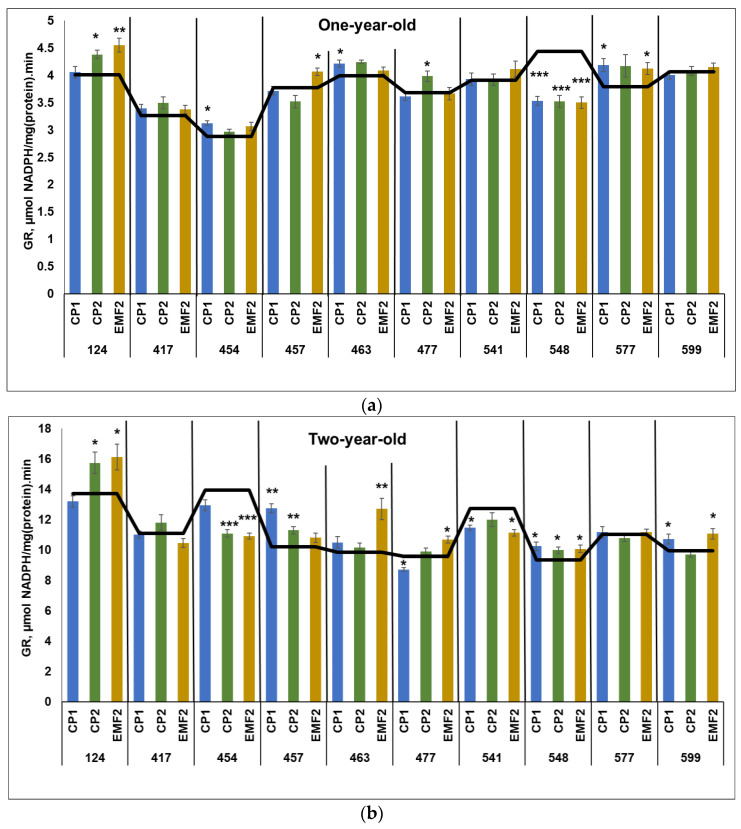
GR activity in needles of (**a**) one-year-old and (**b**) two-year-old seedlings of different half-sib families of *Picea abies* (N = 360; 3 biological replicates × 3 technical replicates for each sample). The black line in the graphs shows the mean values of control; CP1—seed treatment with cold plasma 1 min; CP2—seed treatment with cold plasma 2 min; EMF2—seed treatment with electromagnetic field 2 min. The asterisks indicate the statistical significance of the difference (Student’s test) between the treated group (CP1, CP2, and EMF2) and the control (C) in each half-sib family (* *p* < 0.05; ** *p* < 0.01; *** *p* < 0.001).

**Figure 5:**
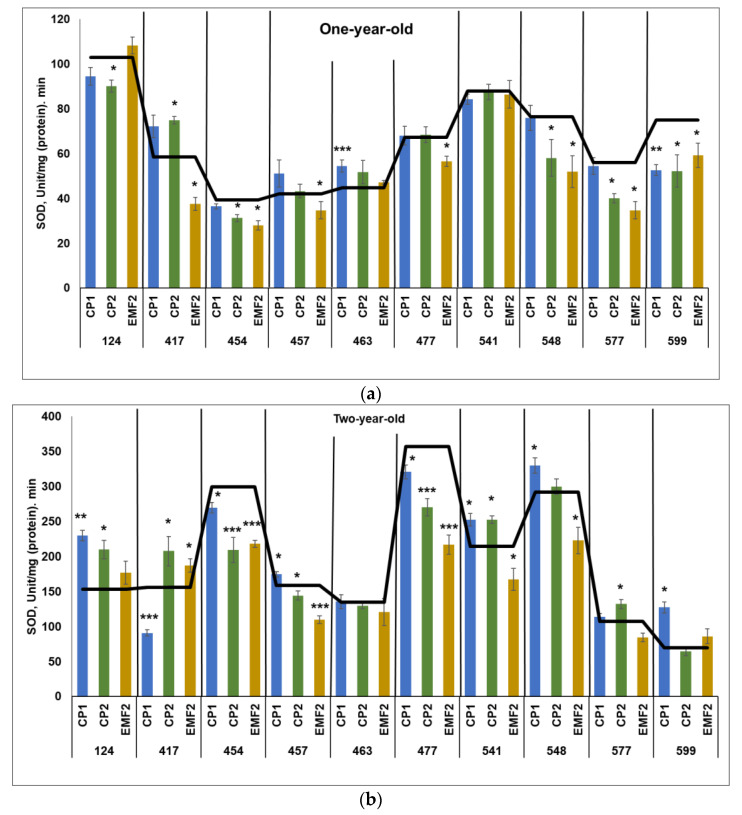
SOD activity in needles of (**a**) one-year-old and (**b**) two-year-old seedlings of different half-sib families of *Picea abies* (N = 360; 3 biological replicates × 3 technical replicates for each sample). The black line in the graphs shows the mean values of control; CP1—seed treatment with cold plasma 1 min; CP2—seed treatment with cold plasma 2 min; EMF2—seed treatment with electromagnetic field 2 min. The asterisks mean the statistical significance of the difference (Student’s test) between the treated group (CP1, CP2, and EMF2) and the control (C) in each half-sib family (* *p* < 0.05; ** *p* < 0.01; *** *p* < 0.001).

The results of the measurement of antioxidant enzyme (CAT, APX, POX, GR, and SOD) activities in *P. abies* needles are presented in Figure 1, Figure 2, Figure 3, Figure 4 and Figure 5.

#### 2.3.1. Catalase (CAT)

The obtained results revealed that CAT activity was three times higher in two-year-old seedlings compared to younger ones, regardless of the half-sib family and the treatment. The highest CAT activity in the control samples in one-year-old seedlings was in the 124, 463, and 599 half-sib families (Figure 1a). Meanwhile, the highest CAT activity in the control samples in two-year-old seedlings was determined in the 124, 454, and 541 half-sib families (Figure 1b).

In one-year-old seedlings, CP1-treatment increased CAT activity in one half-sib family (417) by 27% (Figure 1a). However, the CAT activity in the 124 half-sib family was reduced by 7%. Meanwhile, a longer seed treatment (CP2) resulted in increased CAT activity in three half-sib families (417, 477, and 577) by 27%, 8%, and 13%, respectively, compared to control. However, in the 457 half-sib family CAT activity was reduced by 9% after CP2-treatment. Seed treatment with EMF resulted in enhanced CAT activity in four half-sib families (417, 457, 463, and 577) by 27%, 5%, 8%, and 13%, respectively. The negative effect on CAT activity after seed treatment with EMF was determined in the 548 and 599 half-sib families (the activity was lower by 8% and 12%, respectively). In one family (417), CAT activity was higher after seed treatment with all physical stressors.

In two-year-old seedlings, seed treatment with CP1 increased CAT activity in three half-sib families (457, 463, and 548) by 22%, 14%, and 16%, respectively but reduced by 6% in the 477 half-sib family (Figure 1b). CP2 treatment increased enzyme activity in four half-sib families (124, 417, 457, and 548) by 12%, 14%, 12%, and 16%, respectively. However, in the 454 half-sib family CAT activity was reduced by 15% after CP2-treatment. Meanwhile, EMF-treatment increased CAT activity in six half-sib families –124, 457, 477, 548, and 599 by 8–16%, while in 463 families—by 35%. The negative effect on CAT activity after seed treatment with EMF was observed in the 454 and 541 half-sib families (the activity was lower by 20%). In two half-sib families (457 and 548), CAT activity was higher after seed treatment with all physical stressors, compared to control.

#### 2.3.2. Ascorbate Peroxidase (APX)

The results showed that APX activity was higher in two-year-old seedlings, compared to one-year-old ones, regardless of the half-sib family and treatment. The highest APX activity in the control samples in one-year-old seedlings was determined in the 124, 463, and 541 half-sib families (Figure 2a). Meanwhile, the highest APX activity in the control samples in two-year-old seedlings was observed in the 454 half-sib family (Figure 2b).

In one-year-old seedlings, CP1-treatment increased APX activity in three half-sib families (417, 454, 577) by 22%, 9%, and 9%, respectively (Figure 2a). CP2 treatment resulted in increased APX activity also in three half-sib families (417, 477, and 577) by 19%, 16%, and 13%. However, the APX activity in the 457 half-sib family was reduced by 10%. Meanwhile, EMF-treatment increased APX activity in two half-sib families (417 and 577) by 19% and 10%, respectively. In two half-sib families (417 and 577), APX activity was higher after seed treatment with all physical stressors, compared to control.

Two-years-old seedlings from 457 and 548 half-sib families showed increased APX activity by 19% and 9%, respectively, due to seed treatment with CP1 (Figure 2b). However, the APX activity in the 124 and 477 half-sib families was reduced (less than 10%). Meanwhile, CP2 treatment increased APX activity in three half-sib families (124, 457, and 548) by 12%, 10%, and 9%, respectively. However, in the 454 and 599 half-sib families APX activity was reduced by 20% and 5%, respectively. Seed treatment with EMF increased APX activity in five half-sib families (124, 463, 477, 548, and 599) by 12%, 24%, 9%, 9%, and 8%, respectively. On the contrary, a negative effect of EMF treatment on APX activity was observed in 454 and 541 half-sib families (the APX activity was reduced by 21% and 12%, respectively). In 548 half-sib families only APX activity was higher after seed treatment with all physical stressors, compared to control. Both CP1 and CP2 treatments increased APX activity in the 457 half-sib family.

#### 2.3.3. Peroxidase (POX)

The results indicated that POX activity was higher in two-year-old seedlings compared to younger ones, regardless of the half-sib family and treatment. The highest POX activity in the control samples in one-year-old seedlings was determined in the 457 and 477 half-sib families (Figure 3a). Meanwhile, the highest POX activity in the control samples in two-year-old seedlings was ascertained in the 577 half-sib family (Figure 3b).

In one-year-old seedlings, CP1 treatment increased POX activity in four half-sib families (417, 454, 463, and 577) by 21, 32, 12%, and 15%, respectively (Figure 3a). However, in two families (457 and 477) CP1 reduced POX activity (by 9% and 12%, respectively). Meanwhile, CP2 increased POX activity in five half-sib families (124, 417, 454, 541, and 577) by 20%, 28%, 11%, 19% and 14%, respectively. On the contrary, seed treatment with CP2 reduced POX activity in the 457 half-sib family only (by 12%). Seed treatment with EMF increased POX activity in seven half-sib families (124, 417, 454, 457, 541, 548, and 577) by 16%, 28%, 21%, 5%, 19%, 8%, and 16%, respectively. In three half-sib families (417, 454, and 577), POX activity was higher after seed treatment with all physical stressors, compared to the control.

CP1 treatment increased POX activity in two half-sib families (457 and 548) by 33% and 14%, respectively, in two-year-old seedlings but reduced it by 21% in the 477 half-sib family (Figure 3b). Seed treatment with CP2 led to increased POX activity in the seedling needles from the two half-sib families (124 and 457) by 30% and 10%, respectively. In the two half-sib families (454 and 577) POX activity was reduced by 14% after CP2-treatment. Meanwhile, EMF-treatment increased POX activity in four half-sib families (124, 463, 477, and 548) by 23%, 36%, 15%, and 7%, respectively. However, a negative effect of EMF on POX activity was observed in 454 and 541 half-sib families (POX activity reduced by 21% and 16%, respectively).

#### 2.3.4. Glutathione Reductase (GR)

The results demonstrated that the activity of GR was higher more than 2.5 times in two-year-old seedlings compared to younger ones, regardless of the half-sib family and the treatment. The highest GR activity in the control samples in one-year-old seedlings was determined in the 548 half-sib family (Figure 4a). Meanwhile, the highest GR activity in the control samples in two-year-old seedlings was observed in the 454 half-sib family (Figure 4b).

One-year-old seedlings from 454, 463, and 577 half-sib families showed increased GR activity (by 7%, 8%, and 11%, respectively) due to seed treatment with CP1 (Figure 4a). However, CP1 treatment was negatively affected by 18% GR activity in the 548 half-sib family. CP2 treatment resulted in increased GR activity in two half-sib families (124 and 477) by 8% and 5%, respectively. However, GR activity in 548 half-sib families was reduced by 18%. Meanwhile, seed treatment with EMF increased GR activity in three half-sib families 124, 457, and 577 by 13%, 8%, and 8%, respectively. On the contrary, EMF reduced GR activity in the 548 half-sib family by 18%.

In two-year-old seedlings, CP1-treatment increased GR activity in half-sib families of 457, 548, and 599 by 25%, 9%, and 8%, respectively, but slightly reduced (by less than 10%) GR activity in the 477 and 541 half-sib families (Figure 4b). CP2 increased GR activity in three half-sib families (124, 457, and 548) by 15%, 11%, and 7%, respectively, and reduced it in the 454 half-sib family GR activity by 21%. Meanwhile, EMF-treatment increased GR activity in five half-sib families (124, 463, 477, 548, and 599) by 17%, 28%, 1%, 8%, and 12%, respectively. The negative effect EMF-treatment on GR activity was determined in the 454 and 541 half-sib families (by 21% and 13%, respectively). All physical stressors used for seed treatments induced an increase in GR activity compared to control only in the needles of the 548 half-sib family. Seed treatment with both CP1 and CP2 caused higher GR activity in the 457 half-sib family.

#### 2.3.5. Superoxide Dismutase (SOD)

SOD activity was higher in two-year-old seedlings compared to one-year-old ones, regardless of the half-sib family and the treatment. The highest SOD activity in the control groups of one-year-old seedlings was detected in the 124 half-sib family (Figure 5a), and in the control samples in two-year-old seedlings—in the 477 half-sib family (Figure 5b).

CP-treatment increased SOD activity in one half-sib family (463 after CP1-treatment and 417 after CP2-treatment) by 22% and 28%, respectively in one-year-old seedlings (Figure 5a). However, the SOD activity in the 599 half-sib family was reduced by 30%. In addition, CP2 treatment caused a negative effect in most families—compared to the control, SOD activity was reduced by 13%, 21%, 24%, 28%, and 21% in 124, 454, 548, 577, and 599 half-sib families, respectively. Moreover, EMF-treatment reduced SOD activity in seven half-sib families (417, 454, 457, 477, 548, 577, and 599) by 36%, 29%, 17%, 16%, 32%, 38%, and 21%, respectively.

When SOD activity was assessed in two-year-old seedlings, a more significant impact of seed treatments was found as compared with one-year-old seedlings (Figure 5b). Seed treatment with CP1 enhanced SOD activity in five half-sib families (124, 457, 541, 548, and 599) by 50%, 10%, 18, 13%, and 83%, respectively, although SOD activity in the 417, 454, and 477 half-sib families was reduced by 10%. CP2 increased SOD activity in four half-sib families (124, 417, 541, and 577) by 37%, 33%, 18%, and 13%, respectively, but reduced it in 454, 457, and 477 half-sib families (the activity reduced by 30%, 9%, and 24%, respectively). Meanwhile, EMF-treatment increased SOD activity in the 417 half-sib families by 20%. The negative effect of EMF on SOD activity was observed in the 454, 457, 477, 541, and 548 half-sib families (the activity was lower by 27%, 31%, 39%, 22%, and 24%, respectively). In two half-sib families (124 and 541) SOD activity was increased by both CP1 and CP2 treatments.

### 2.4. Changes of Antioxidant Activity in P. abies Needles

The results of the conducted measurements of antioxidant activity (DPPH and ABTS) in *P. abies* needles are presented in Table 3.

**Table 3:** Radical scavenging activity in needles of (**a**) one-year-old and (**b**) two-year-old seedlings of different half-sib families of *Picea abies*. C—control; CP1—seed treatment with cold plasma 1 min; CP2—seed treatment with cold plasma 2 min; EMF2—seed treatment with electromagnetic field 2 min. The asterisks indicate the statistical significance of the difference (Student’s test) between the treated group (CP1, CP2, and EMF2) and the control (C) in each half-sib family (* *p* < 0.05; ** *p* < 0.01; *** *p* < 0.001).

(a)								
Half-Sib Family	Radical Scavenging Activity (µmol/g) ± SE One-Year-Old							
	DPPH Assay				ABTS Assay			
	C	CP1	CP2	EMF2	C	CP1	CP2	EMF2
124	78.0 ± 5.7	81.3 ± 8.5	75.8 ± 10.6	76.3 ± 6.6	591.4 ± 24.3	574.6 ± 22.6	629.7 ± 18.0	606.8 ± 14.8
417	86.9 ± 9.2	72.8 ± 11.7	51.3 ± 7.1 *	90.5 ± 8.8	790.9 ± 21.9	734.5 ± 14.6 *	642.9 ± 20.6 ***	805.6 ± 33.8
454	78.0 ± 7.6	63.3 ± 6.8	61.9 ± 13.6	69.7 ± 7.7	471.7 ± 12.2	517.4 ± 18.6 *	586.2 ± 30.4 **	639.0 ± 22.4 ***
457	72.8 ± 6.3	88.4 ± 8.2	85.7 ± 6.3	80.4 ± 7.3	610.9 ± 26.9	641.0 ± 6.1	616.2 ± 17.9	549.4 ± 21.4
463	72.6 ± 6.6	75.8 ± 12.2	93.0 ± 7.6 *	92.3 ± 6.0 *	539.0 ± 10.6	635.6 ± 11.2 ***	622.0 ± 13.3 ***	592.4 ± 10.0 *
477	71.1 ± 10.7	71.5 ± 6.1	65.2 ± 8.9	70.8 ± 8.2	475.6 ± 20.1	462.2 ± 18.2	534.4 ± 33.5	579.8 ± 10.7 ***
541	68.2 ± 8.8	48.7 ± 6.7	68.8 ± 5.8	65.0 ± 6.4	712.8 ± 81.9	573.9 ± 17.6	695.6 ± 12.2	613.3 ± 12.7
548	83.9 ± 4.7	64.7 ± 6.4 *	75.1 ± 5.3	78.5 ± 6.0	559.9 ± 12.9	549.5 ± 22.7	616.5 ± 14.2 *	628.2 ± 19.3 *
577	80.8 ± 7.3	104.2 ± 6.6 *	95.0 ± 7.2	89.0 ± 9.6	614.7 ± 17.2	576.3 ± 16.0	600.0 ± 12.1	596.0 ± 20.5
599	73.4 ± 12.0	81.0 ± 10.7	89.5 ± 6.1	83.2 ± 7.6	733.1 ± 26.1	803.8 ± 30.0	718.1 ± 18.6	662.3 ± 22.8 *
**(b)**								
**Half-Sib Family**	**Radical Scavenging Activity (µmol/g) ± SE** **Two-Year-Old**							
	**DPPH Assay**				**ABTS Assay**			
	**C**	**CP1**	**CP2**	**EMF2**	**C**	**CP1**	**CP2**	**EMF2**
124	587.8 ± 13.3	633.9 ± 9.7 *	560.6 ± 10.6	601.7 ± 20.8	1418.3 ± 24.8	1397.7 ± 29.1	1206.2 ± 27.6 ***	1243.1 ± 25.0 ***
417	650.6 ± 10.7	599.9 ± 14.9 *	614.6 ± 9.8 *	681.4 ± 14.8	1402.0 ± 20.9	1322.1 ± 24.0 *	1324.0 ± 17.1 *	1468.6 ± 31.4
454	563.4 ± 19.5	579.3 ± 11.6	559.6 ± 9.0	542.8 ± 14.2	1117.1 ± 14.3	1199.0 ± 35.6 *	1099.5 ± 17.6	1094.6 ± 23.4
457	559.2 ± 16.7	616.4 ± 18.1 *	712.2 ± 19.1 ***	660.5 ± 10.8 ***	1283.1 ± 22.6	1364.1 ± 28.4 *	1510.3 ± 40.5 ***	1375.8 ± 22.9 *
463	584.6 ± 13.4	569.8 ± 15.7	613.1 ± 16.3	566.9 ± 16.6	1053.4 ± 17.2	1122.9 ± 21.4 *	1230.4 ± 32.7 ***	996.8 ± 20.9 *
477	617.6 ± 12.2	668.0 ± 20.1 *	594.7 ± 12.2	774.0 ± 43.6 **	1339.7 ± 23.2	1366.1 ± 27.5	1169.3 ± 36.3	1557.1 ± 83.8
541	581.9 ± 13.4	605.1 ± 14.8	537.9 ± 10.0 *	559.7 ± 10.4	1159.1 ± 24.5	1140.1 ± 19.1	1203.1 ± 26.6	1375.6 ± 43.3 ***
548	586.7 ± 15.1	537.5 ± 14.4 *	546.2 ± 12.8 *	573.7 ± 20.3	1125.3 ± 42.5	1097.4 ± 27.2	1056.2 ± 39.0	1079.3 ± 37.4
577	557.7 ± 13.3	551.2 ± 12.6	626.1 ± 20.8 *	469.4 ± 13.2 ***	1058.4 ± 13.5	1035.4 ± 26.0	1064.5 ± 28.6	796.1 ± 17.1 ***
599	662.0 ± 16.1	607.5 ± 17.0 *	678.3 ± 11.7	660.3 ± 17.9	1409.9 ± 35.7	1266.1 ± 33.1 **	1308.5 ± 22.9 *	1367.4 ± 19.7

Radical scavenging activity in the different half-sib families of *P. abies* needles was determined using two tests: DPPH (2,2-diphenyl-1-picryl-hydrazylhydrate) and ABTS (2,2′-azino-bis(3-ethylbenzothiazoline-6-sulfonic acid) assays. The difference in antioxidant activity in needles between one-year-old and two-year-old seedlings in the same experimental group was statistically significant differences (*p* < 0.05) in all half-sib families. The antioxidant activity was higher eight times (DPPH) and two times (ABTS) in two-year-old seedlings compared to one-year-old seedlings (Table 3). In the control samples, the highest antioxidant activity in one-year-old seedlings (86.93 µmol/g (DPPH) and 790.86 µmol/g (ABTS), respectively) was observed in the 417 half-sib family, while the other two families exhibited the highest values in two-year-old seedlings 599 (661.96 µmol/g (DPPH) and 124 (1418.29 µmol/g (ABTS) half-sib families (Table 3). The antioxidant activity in groups of seedlings growing from treated seeds was also dependent on the half-sib family, and different families exhibited diverse responses to the used physical stressors and treatment duration.

Seed treatment with CP1 increased antioxidant activity (DPPH assay) only in the 577 half-sib family (activity was higher by 29%) but reduced the antioxidant activity in the 548 half-sib family by 23% in one-year-old seedlings (Table 3a). However, CP2 treatment increased activity (by 28%) only in the 463 half-sib family, and a similar trend was observed after seed treatment with EMF. Compared to DPPH, different changes were revealed using ABTS assay. In one-year-old seedlings, seed treatment with CP1 increased antioxidant activity in two half-sib families (454 and 463 by less than 20%) and reduced it in 417 half-sib families. Meanwhile, CP2-treatment increased antioxidant activity in three half-sib families 454, 463, and 548 by 24%, 15%, and 10%, respectively. The activity was reduced in 417 half-sib families, as in the case with CP1 treatment. EMF-treatment increased antioxidant activity in the 454, 463, 477 and 548 half-sib families (by 35%, 10%, 22%, and 12%, respectively), but reduced it by less than 10% in the 599 half-sib family. In two families (454 and 463) antioxidant activity (using the ABTS method) was higher after seed treatment with all protocols.

The differences in ABTS activity in one half-sib family (541) increased by 36% in the CP1-treated group, compared to the control (Table 3a). In addition, the differences in ABTS activity in the 599 half-sib family were reduced by 35% in the CP1-treated group, compared to the control. The differences in ABTS activity in the 457 half-sib family increased by 35% in the CP2-treated group, compared to the control. Meanwhile, the differences in ABTS activity in three half-sib families (124, 454, and 477) reduced by 48%, 49%, and 63%, respectively in the CP2-treated group, compared to the control. On the other hand, two half-sib families (457 and 541) increased in differences of ABTS activity by 40% and 62%, respectively in the EMF-treated group, compared to the control. However, the differences in ABTS activity in three half-sib families (124, 454, and 577) were reduced by 35%, 66%, and 39%, respectively compared to the control groups.

Seed treatment with CP1 resulted in increased antioxidant activity in two-year-old seedlings (using DPPH assay) in three half-sib families (124, 457, and 477) by 8%, 10%, and 8%, respectively (Table 3b). However, seed treatment with CP1 reduced antioxidant activity in 417, 548, and 599 half-sib families by 8%. Meanwhile, CP2-treatment increased antioxidant activity in two half-sib families (457 and 577 by 27% and 12%, respectively), but reduced antioxidant activity by less than 10% in three half-sib families (417, 541, and 548). EMF-treatment resulted in higher antioxidant activity in two half-sib families (457 and 477 by 18% and 25%, respectively) and reduced antioxidant activity in the 577 half-sib family by 19%. In one family (457) antioxidant activity (using the DPPH method) was higher after seed treatment with all physical stressors.

It was observed using ABTS assay, that antioxidant activity was higher (by less than 10%) in the 454, 457, and 463 half-sib families (Table 3b). However, CP1 treatment reduced antioxidant activity in 417 and 599 half-sib families (by less than 10%). Seed treatment with CP2 caused an increase in antioxidant activity in the 457 and 463 half-sib families by 17%. However, in the 124, 417, and 599 half-sib families activity was reduced (by less than 15%). EMF-treatment increased antioxidant activity in 457 and 541 half-sib families by 7% and 17%, respectively, and reduced it in three half-sib families (124, 463, and 577 by 12%, 5%, and 25%, respectively).

### 2.5. Heatmap, Correlation, and Principal Component Analysis (PCA) between Antioxidant Enzymes and Antioxidant Activity

MANOVA analysis showed that the age of seedlings, different half-sib families, and the treatment had a significant influence on *P. abies* antioxidant system (enzymes, DPPH, and ABTS) with the largest impact by an age of seedlings (Wilks lambda—0.02, *p* < 0.001).

**Figure 6:**
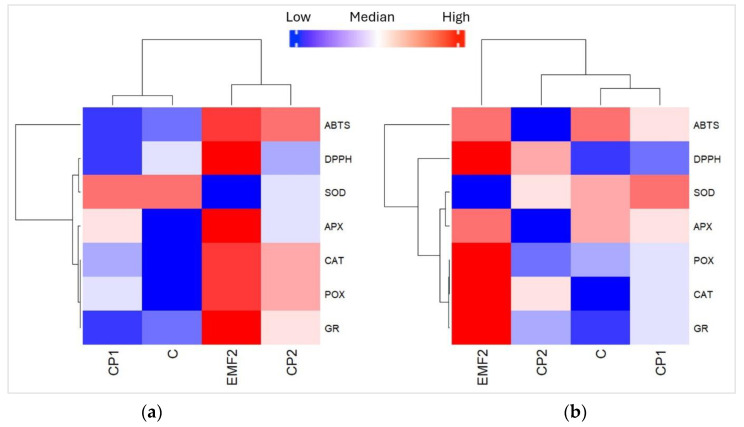
Heatmap for different enzymes and antioxidant activity of needles from (**a**) one-year-old and (**b**) two-year-old *Picea abies* seedlings growing from the seeds treated by CP1, CP2, and EMF2. Control (C) represents needles from seedlings growing from non-treated seeds.

**Figure 7:**
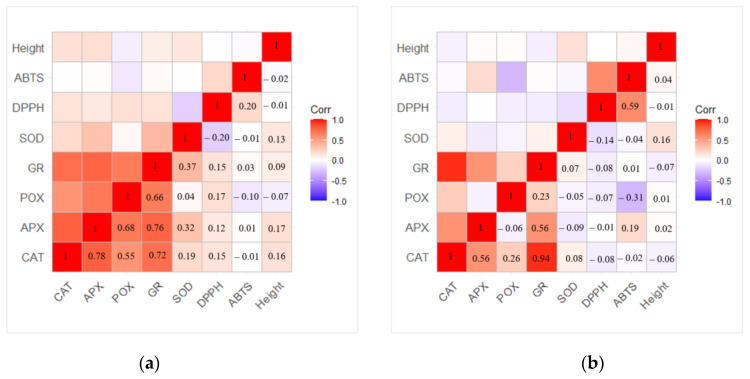
Matrix of correlation between different variables in (**a**) one-year-old and (**b**) two-year-old *Picea abies* seedlings based on the Pearson correlation coefficient.

Heatmaps display the variation in the content of enzymes (CAT, APX, POX, GR, and SOD) and antioxidant activity (ABTS and DPPH) in the needles of one-year-old (Figure 6a) and two-year-old (Figure 6b) seedlings in groups growing from the seeds treated with physical stressors (CP1, CP2, and EMF). Blue colors indicate that the value of the analyzed variable was below the mean level, while red colors indicate values higher than the mean. For one-year-old seedlings, results obtained for seedlings growing from untreated seeds (C) and those treated with CP1, CP2, and EMF were clustered into two groups: the first cluster contained CP1 and C samples, while the second cluster contained EMF and CP2 (Figure 7a). The analysis shows that POX activity was significantly higher (*p* < 0.05) in the needles from one-year-old seedlings growing from seeds treated with EMF, compared to the control group. In contrast, EMF decreased the activity of SOD. Analyzing two-year-old seedlings, the results obtained from C, CP1, CP2, and EMF2 groups were clustered into three groups, where the first cluster contained C and CP1, the second—included CP2, and the third—included EMF (Figure 6b). The EMF-induced changes in the activity of POX and SOD followed the same trend as in one-year-old seedlings. In addition, EMF increased the activity of CAT in the needles of the two-year-old seedlings.

The correlation analysis revealed a strong positive dependence between activities of GR, POX, APX, and CAT antioxidant enzymes, especially between GR—CAT (r = 0.72) and GR—APX (r = 0.76) in one-year-old seedlings (Figure 7a). Meanwhile, a moderate correlation between SOD—APX and SOD—GR was obtained (r = 0.32 and r = 0.37, respectively). The strongest correlation in two-year-old seedlings was determined between GR—CAT enzymes (r = 0.94) (Figure 7b). The correlation between SOD and other enzymes was not determined. In addition, differences between changes in POX activity compared to changes in DPPH or ABTS scavenging were determined. The moderate negative correlation between ABTS—POX (r = −0.31) was observed, while the correlation between DPPH—POX (−0.07) was not observed.

**Figure 8:**
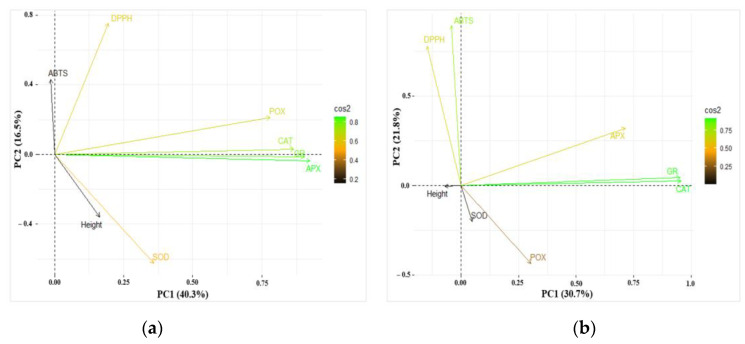
Principal component analysis (PCA) displaying the first and second components (PCs) from the data of (**a**) one-year-old and (**b**) two-year-old *Picea abies* seedlings. The loading vectors show the correlation between different variables. Colors of vectors from black to green represent different squared cosine (Cos2) values.

The results of PCA for data obtained on height, enzyme (CAT, APX, POX, GR, and SOD) activities, and antioxidant activity (ABTS and DPPH assays) in the needles of one-year-old and two-year-old are presented in Figure 8a and Figure 8b, respectively. The first four of the seven principal components explained 82.3% of the total variance of the parameters, while the first two—56.9% in one-year-old seedlings (Table 4). Similarly, in two-year-old seedlings, the first four principal components explained 79.5% of the total variance of the parameters, while the first two—52.5% SOD was negatively linked to PC2 in one-year-old seedlings, while ABTS and DPPH were positively linked to PC2 (Table 5). The enzymes CAT, APX, POX, and GR were grouped together and positively linked to PC1. Analyzing two-year-old seedlings, similarly to one-year-old seedlings, ABTS and DPPH were positively linked to PC2. However, POX was negatively linked to PC2 in two-year-old seedlings. Analyzing the results of PCA for data with the treatments separately, it was found that CAT and GR were grouped together and positively linked to PC1 after all the treatments in both one-year-old and two-year-old seedlings (Supplementary Figures S1 and S2). APX was grouped together with CAT and GR after all the treatments in one-year-old seedlings as well, however, in two-year-old seedlings, APX was grouped together with CAT and GR only after the treatment with CP1, the same as in the control. ABTS and DPPH were positively linked to PC2 in two-year-old seedlings after the treatments with CP1 and EMF2, the same as in the control (Supplementary Figures S1 and S2).

## 3. Discussion

During the long lifespans of gymnosperms, they are attacked by different pests and pathogens [45], and regularly face drought and other stressful environmental conditions [46]. To cope with potential damages induced by numerous biotic and abiotic factors, the gymnosperms have evolved many constitutive and inducible mechanisms, including the production of defensive enzymes, which act across different lifespans and types of tissues [45,47,48,49,50].

Pre-sowing plant seed treatment with cold plasma (CP) and electromagnetic field (EMF) has been proved as potential stressors eliciting complex plant response that can result in improvement of agricultural performance and stress resistance of numerous crops [51,52]. It was demonstrated that moderate stress experienced by the plasma or EMF-irradiated seed can lead to a multifaceted long-term response in the growing plant, including activated photosynthesis, secondary metabolism, and increased harvest yield. Although numerous studies have reported changes induced in the activity of the antioxidant defense system, the obtained results have been controversial [53].

Pre-sowing seed treatment with CP and EMF showed a potential to increase the resistance of forest trees as well [17,18,54,55]. However, numerous knowledge gaps related to changes induced in biochemical and physiological processes and antioxidant defense capacity in perennials remain unresolved. Thus, the purpose of this study was to compare the effects of pre-sowing treatment of *P. abies* seeds with CP and EMF on different half-sib families by observing changes in seedling emergence and growth of one-year-old and two-year-old seedlings, with particular attention to changes in the antioxidant system in seedling needles.

No significant effect of seed treatments with CP1, CP2, and EMF on the emergence of *P. abies* seedlings was observed in our study. These results confirmed earlier reported observations that the seed treatment with CP and EMF may not have a significant impact on the emergence of gymnosperm seedlings [56,57,58,59]. However, we found that pre-sowing seed treatment with physical stressors might cause a stronger impact on growth (estimated by changes in the height) of one-year-old seedlings compared to two-year-old *P. abies* seedlings. The treatment with CP1 and CP2 had a positive effect on 50% and 40% of half-sib families, respectively, while EMF had a negative effect on 40% of one-year-old half-sib families. Meanwhile, the significant impact of these treatments on the height of two-year-old seedlings was not determined. Competition among trees is an important driver of community structure in forests, and our results indicated that the pre-sowing seed treatments may impact tree seedling establishment and early growth [21,59].

The obtained results also demonstrated evidence that pre-sowing seed treatments with CP and EMF had the potential to cause significant changes in metabolic processes in *P. abies* seedlings. Catalytic proteins play an essential role in managing and stabilizing reactive oxygen species (ROS) [37]. Under environmental stress conditions, the level of ROS that causes oxidative cell damage in plants could be buffered by enzymatic antioxidants [60,61]. It was demonstrated that the accumulation of biologically active compounds in conifers depends on the tree’s age [62]. CP and EMF-induced changes in biochemical plant protection systems are age-dependent, as highlighted by higher amounts of chlorophylls and phenols in two-year-old than one-year-old *P. abies* seedlings [21]. The results of this study supplement such findings indicating that the activity of antioxidant enzymes is significantly higher in two-year-old than one-year-old seedlings.

One of the enzymes is superoxide dismutase (SOD), which is responsible for scavenging free radicals, particularly superoxide anion •O_2_^−^, produced during photosynthesis and other metabolic processes [38,63]. Our study showed that, unlike other enzymes, seed treatment with CP2 and EMF might cause a decrease in SOD activity in one-year-old seedlings. Meanwhile, the treatment with CP1 has the potential to increase SOD activity in two-year-old seedlings. The increase in SOD activity is primarily related to plant response to drought [64,65,66] and salinity [38,67]. Pre-sowing seed treatment with CP and EMF may have either a positive or negative effect on SOD activity, dependent on the half-sib family. Whereas SOD converts superoxide anions into H_2_O_2_, which is dangerous for plant cells, other enzymes, such as CAT, APX, POX, and GR operate in tight cooperation with SOD, since they neutralize the toxic H_2_O_2_ or restore GSH from oxidated GSSG, preventing the Haber-Weiss reaction or the Foyer–Halliwell–Asada pathway [38]. Although the reaction mechanisms of CAT/POX, APX, and GR are different, they function as elements of a single defense system against biotic and abiotic stress [38]. In our study, a strong correlation between CAT, APX, POX, and GR activities was also determined. According to other studies, water stress can increase CAT, POX, APX, and GR enzyme activity in *Cucurbita pepo* [68]. A similar increase in CAT and POX activities has been reported after chemical stress (various solutions of NaCl) in *Berseem clover* [69]. Furthermore, the application of biological treatment (jasmonic acid) can increase either CAT [70] or POX [71] activity in plants. The treatment with a magnetic field can also activate the synthesis of SOD and CAT enzymes in soybeans, indicating a strong relationship between their activities [72]. Our study provides evidence that pre-sowing seed treatment with CP and EMF can induce multiple changes in the activities of antioxidant enzymes; however, these changes were influenced by the treatment and *P. abies* half-sib family. For instance, in one-year-old seedlings of the 577 half-sib family, the activity of CAT, APX, POX, and GR increased after EMF-treatment, compared to the control group, while in the 477 half-sib family, CAT, APX, and GR activities increased after CP2-treatment. In two-year-old seedlings of the 548 half-sib family, the activity of CAT, APX, POX, and GR increased with the pre-sowing seed treatment with EMF. In the half-sib families 548 and 463 the activity of CAT, APX, POX, and GR had the potential to increase after pre-sowing treatment with CP1 and EMF, respectively. In the 457 half-sib family, none of the analyzed parameters (except height) changed after CP1-treatment in one-year-old seedlings.

The free radical scavenging capacity in plant extracts is commonly based on DPPH and ABTS tests [73]. It was determined that seed treatment with a magnetic field increased antioxidant activity in different sunflower extracts; however, this effect was dependent on plant variety [74]. Seed treatment with bio-stimulants enhanced ROS scavenging activity in young olive trees [75]. Pre-sowing treatment of *P. abies* seeds with CP and EMF also promises to increase radical scavenging activity in the needles of one-year-old and two-year-old seedlings, but it depends on the half-sib family. The results showed that both CP and EMF increased radical scavenging activity (ABTS and DPPH) in the 457 half-sib family of one-year-old and two-year-old seedlings. The differences in changes in antioxidant activity (estimated by both ABTS and DPPH assays) between the treatments with CP1 and CP2 were also observed.

In conclusion, pre-sowing seed treatment with CP and EMF has the potential to induce significant changes in the activities of antioxidant enzymes and radical scavenging in one-year-old and two-year-old *P. abies* seedlings, without significant impact on seedling emergence and height. The impact of pre-sowing seed treatment with CP on biochemical processes depended on the duration of treatment (1 min and 2 min). The changes induced in the activity of enzymes and radical scavenging activity were specific to different half-sib families. These treatments can be promising to apply, but for practical use, hormones and plant gene expression or other factors must be analyzed. To evaluate the impact of physical stress experienced by seeds on different *P. abies* half-sib families, further long-term observations are needed. Furthermore, to assess the potential application of pre-sowing seed treatments involving CP and EMF, it is necessary to test experimental models measuring changes in the antioxidant system following biotic and abiotic stress, such as drought, salinity, or attacks by pests and/or pathogens.

## 4. Materials and Methods

### 4.1. Seed Collection and Treatment with Cold Plasma (CP) and Electromagnetic Field (EMF)

Ten different half-sib families of *P. abies* seeds were collected from a second-generation spruce seed orchard in the Trakai regional division (Lithuania) (Table 6). The collected seeds were kept in refrigeration until the treatment. Seeds were divided into four different groups: control; seed treatment with cold plasma for 1 min (CP1), seed treatment with cold plasma for 2 min (CP2), and seed treatment with electromagnetic field for 2 min (EMF2). The total amount of seeds in experiments was 6400 (10 half-sib families × 4 treatment protocols × 160 seeds per treatment).

*Seed treatment with cold plasma (CP).* The seeds of each *P. abies* half-sib family were irradiated using a low-temperature atmospheric dielectric barrier discharge (DBD) plasma device produced at Kyushy University, Fukuota, Japan [76]. The treatment was performed at Vytautas Magnus University, Kaunas, Lithuania. Seeds were placed on a glass plate in an area of homogeneous treatment (4 × 4.38 cm^2^) at a distance of 5 mm between the seed surface and the electrode plate; the discharge voltage was 7.0 kV, the current was 0.2 A; the power density was 3.1 W/cm^2^; the humidity was 45–55%; the atmospheric pressure and room temperature [17]. The duration of treatment was 1 and 2 min.

*Seed treatment with electromagnetic field (EMF).* The seeds of each *P. abies* half-sib family were exposed to radiofrequency electromagnetic field (EMF) treatment with a pulsed magnetic field generator designed at Vilnius Gediminas Technical University (VilniusTECH), Lithuania. The seeds were treated for 2 min (parameters: 100 kHz, 400 ± 50 μT oscillating magnetic field; atmospheric pressure and room temperature) [18]. The magnetic field was measured using a calibrated loop sensor (VilniusTECH, Vilnius, Lithuania), and a DPO4034 oscilloscope (Tektronix, Beaverton, OR, USA). The peak amplitude value was also confirmed using a Gaussmeter 475DSP (Lakeshore, Carson, CA, USA).

### 4.2. Seedling Cultivation

**Figure 9:**
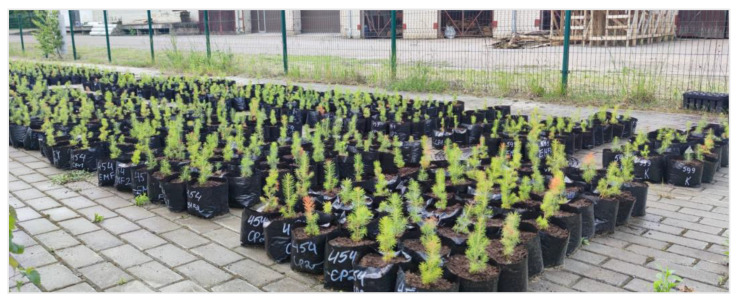
Treated and control one-year-old *Picea abies* seedlings in pots.

Treated *P. abies* seeds were stored in plastic bags for 4 days at 25 °C, to allow sufficient time for alterations in the levels of phytohormones within the seeds [27]. The treated and control seeds were sown in the cassettes (40-cell) with a peat substrate (SuliFlor SF2, pH of 5.5–6.5) and lightly covered with perlite. The seedlings were cultivated in a controlled-condition greenhouse (25–32 °C daytime; >10 °C at night) for two months. Then the seedlings were transferred to an open area. After a year, the seedlings were transplanted into larger pots (18 × 17 × 2 cm) filled with 1000 mL of peat substrate (Figure 9).

### 4.3. Seedling Emergence and Height

The emergence of *P. abies* seedlings started to count one week after the sowing and continued for 15 days. The percentage of emerged seedlings along with the standard error was calculated [18]. *P. abies* seedlings height measurement (cm) was completed after the vegetation period in both one-year-old and two-year-old seedlings.

### 4.4. Sample Collection

The samples of *P. abies* needles were collected (three biological replicates) both from seedlings in the control and treated groups at the end of the first and the second vegetation season. The needle samples were selected to collect from random seedlings by taking approximately 30 needles from different analyzed groups. Fresh needle samples were partitioned into equal weight and stored at −20 °C until the start of the biochemical analyses.

### 4.5. Sample Preparation for Antioxidant Enzymes Analysis

*P. abies* needle extracts were prepared for the assessments using the method described by [18]. 0.2 g needles were powdered in liquid nitrogen and transferred to 15 mL tubes, and 5 mL of extraction buffer (containing K-phosphate buffer (pH 7.8), 1% [*v*/*v*] Triton X-100, 300 mg polyvinylpolypyrrolidone (PVPP) and 5 mM ascorbate (ASC)) solution was added. The mixture with the sample was centrifuged for 1 h, 16,090× *g*, +4 °C (Andreas Hettich GmbH & Co. KG, Tuttlingen, Germany). The supernatant was promptly transferred to 96 microplates for assessing the overall protein content (PROT) and measuring the activities of superoxide dismutase (SOD) and catalase (CAT). Separation of the supernatant and extract for the assessment of ascorbate peroxidase (APX), peroxidase (POX), and glutathione reductase (GR) was completed using Sephadex G-25 (Column PD-10, Cytiva, Gillingham, UK) columns on ice.

### 4.6. Protein Measurement and Kinetic Assays of Antioxidant Enzymes: CAT, APX, POX, GR, and SOD

All kinetic assays were performed in a 96-well microplate setup (Greiner Bio-One GmbH, Frickenhausen, Germany) using a SpectroStar Nano microplate reader (BMG Labtech, Offenburg, Germany), extract volume (20 µL), and a total assay (reaction buffer) volume (170–180 µL) in each well. Evaluation of total protein content in the needle extract was performed according to Čėsnienė et al., 2023 [18] methodology. The reaction mixture consisted of Biuret reagent and Folin–Ciocalteu reagent (1:9 *w*/*v*). The absorbance was measured at a wavelength of 660 nm. As a standard it was used Bovine Serum Albumin (BSA). The protein content in the crude extract was measured in mg/mL (the total amount of mg equivalent to BSA per milliliter).

Measurement of CAT, APX, POX, GR, and SOD activity in the needle extracts was performed according to Čėsnienė et al., 2023 [18]. The absorbance was measured at a wavelength of 240 nm, 290 nm, 430 nm, 340 nm, and 550 nm, respectively. Protein concentration in the fresh needles is used for the assessment of CAT, APX, and POX activity that was expressed in µmol H_2_O_2_ mg protein^−1^ min^−1^ in the needle biomass, as described by Wu and von Tiedeman, 2002 [76] methodology. Based on the protein concentration in the fresh needle tissue, the GR activity was measured using the oxidation of NADPH reducing oxidized L-glutathione reaction, expressed in μmol GSSG mg protein^−1^ min^−1^ in the needle biomass as described by Wu and von Tiedeman, 2002 [77] methodology. SOD activity was measured using nitroblue tetrazolium (NBT) reduction, expressed in units mg protein^−1^ min^−1^ in the needle biomass, according to Dhindsa et al., 1981 [78] methodology.

### 4.7. Sample Preparation for Antioxidant Activity Analysis

For detection of the antioxidant activity, both DPPH (2,2-diphenyl-1-picryl-hydrazyl-hydrate) and ABTS (2,2′-azino-bis(3-ethylbenzothiazoline-6-sulfonic acid) radical scavenging tests were used according to Čėsnienė et al., 2023 [18] methodology. Needle samples (0.5 g) were homogenized, and mixed with MeOH (75%), and extracts were shaken at room temperature for 24 h, using a Kuhner Shaker X electronic shaker. After a 24-h period, the needle extracts underwent purification using Rotilabo^®^-113A (Ø 90 mm) filter papers.

### 4.8. Antioxidant Activity Test: DPPH and ABTS Radical Scavenging

Free radical scavenging activity was determined by DPPH assay in MeOH extracts of *P. abies* needle samples as described by Ragaee et al., 2006 [79] methodology. The DPPH reaction solution was mixed with MeOH and extract and transferred to dark for 16 min. The absorbance was measured at a wavelength of 515 nm. The DPPH radical scavenging activity was calculated as antioxidant Trolox equivalents/per gram of fresh needle material [18].

Free radicals scavenging activity was determined by ABTS assay in MeOH extracts of *P. abies* needle samples according to Lučinskaitė et al., 2021 [80] methodology. The ABTS reaction solution was mixed with extract and transferred to dark for 10 min. The absorbance was measured at a wavelength of 734 nm. The radical scavenging activity using ABTS assays was calculated the same as DPPH (see description above).

### 4.9. Statistical Analysis

Statistical analysis was performed using R (Version 4.3.1) with RStudio (Version 2023.06.0) and Microsoft Excel 2010. Column charts of antioxidant enzymes with error bars represented the mean values along with the standard error (SE), while the emergence rate, seedling height, and antioxidant system (DPPH and ABTS) were presented as means in the tables (±SE). The statistically significant differences between the two groups (control and treatment: CP1, CP2, and EMF2) were calculated using Student’s *t* test [81]. The Benjamini and Hochberg method was used for the adjustment of *p*-values for multiple comparisons [82]. In order to determine whether tested factors (treatment, year, half-sib family) had a significant influence on *Picea abies* antioxidant system, MANOVA was performed [83]. To compute the correlation matrix of the different components of the antioxidant system, the *cor* function from the *corrr* package was used [84]. For the visualization, the *ggcorplot* function from the *ggplot2* package was applied [85]. To visualize the outputs of the principal component analysis (PCA) of the components of the antioxidant system, the *princomp*, *summary, fviz_eig*, *fviz_pca_var*, and *fviz_cos2* functions from the *FactoMineR* package were applied [86]. Numeric differences in the mean values of different antioxidant components with different colors were visualized using the *heatmap* function.

## Figures and Tables

**Table 1:** *Picea abies* emergence percentage (%) in different half-sib families. C—control; CP1—seed treatment with cold plasma 1 min; CP2—seed treatment with cold plasma 2 min; EMF2—seed treatment with electromagnetic field 2 min. The asterisks indicate the statistical significance of the difference (Student’s test) between the treated group (CP1, CP2, and EMF2) and the control (C) in each half-sib family (* *p* < 0.05; ** *p* < 0.01; *** *p* < 0.001).

Half-Sib Family	Emergence (%) ± SE			
	C	CP1	CP2	EMF2
124	68.1 ± 0.4	65.0 ± 0.4	61.3 ± 0.4	68.8 ± 0.4
417	87.5 ± 0.3	79.4 ± 0.3 *	85.0 ± 0.3	71.9 ± 0.3 ***
454	79.4 ± 0.3	55.6 ± 0.4 ***	60.0 ± 0.4 ***	66.3 ± 0.4 **
457	43.1 ± 0.4	52.5 ± 0.4	48.1 ± 0.4	52.5 ± 0.4
463	66.7 ± 0.6	63.9 ± 0.6	75.0 ± 0.5	80.6 ± 0.3 *
477	46.3 ± 0.4	52.5 ± 0.4	62.5 ± 0.4 **	55.0 ± 0.4
541	69.4 ± 0.4	63.8 ± 0.4	57.5 ± 0.4 *	59.4 ± 0.4
548	61.3 ± 0.4	67.5 ± 0.4	61.9 ± 0.4	63.2 ± 0.4
577	65.6 ± 0.4	58.8 ± 0.4	54.4 ± 0.4 *	60.0 ± 0.4
599	35.6 ± 0.4	35.6 ± 0.4	39.4 ± 0.4	31.3 ± 0.4

**Table 2:** Height of one-year-old and two-year-old seedlings in different *Picea abies* half-sib families. C—control; CP1—seed treatment with cold plasma 1 min; CP2—seed treatment with cold plasma 2 min; EMF2—seed treatment with electromagnetic field 2 min. The asterisks indicate the statistical significance of the difference (Student’s test) between the treated group (CP1, CP2, and EMF2) and the control (C) in each half-sib family (* *p* < 0.05; ** *p* < 0.01; *** *p* < 0.001).

Half-Sib Family	Height (cm)							
	One-Year-Old				Two-Year-Old			
	C	CP1	CP2	EMF2	C	CP1	CP2	EMF2
124	7.6 ± 0.2	7.6 ± 0.3	7.3 ± 0.2	7.0 ± 0.3	12.5 ± 0.6	13.1 ± 0.9	13.0 ± 0.5	11.9 ± 0.5
417	5.9 ± 0.2	6.9 ± 0.2 **	6.2 ± 0.2	5.1 ± 0.2 **	10.6 ± 0.5	13.3 ± 0.5 ***	10.4 ± 0.9	9.9 ± 0.7
454	8.7 ± 0.3	7.5 ± 0.4 *	7.3 ± 0.2 ***	7.7 ± 0.2 *	15.4 ± 0.7	14.0 ± 0.7	14.4 ± 0.5	13.7 ± 0.6
457	6.7 ± 0.3	7.9 ± 0.2 ***	7.9 ± 0.2 ***	7.6 ± 0.3 *	15.0 ± 0.8	17.0 ± 0.9 *	15.5 ± 0.6	15.2 ± 0.8
463	5.3 ± 0.3	8.3 ± 0.4 ***	6.3 ± 0.3 *	5.3 ± 0.2	17.1 ± 1.5	16.6 ± 1.2	13.5 ± 0.9 *	15.2 ± 1.3
477	5.8 ± 0.3	6.9 ± 0.3 *	6.2 ± 0.3	5.6 ± 0.3	14.6 ± 0.8	16.5 ± 0.9	13.7 ± 1.2	13.9 ± 0.9
541	8.8 ± 0.3	8.8 ± 0.3	8.1 ± 0.3	7.9 ± 0.3 *	14.1 ± 0.4	15.7 ± 0.6 *	13.8 ± 0.9	12.7 ± 0.6 *
548	6.7 ± 0.2	7.9 ± 0.2 ***	8.7 ± 0.2 ***	6.8 ± 0.2	15.0 ± 0.8	15.8 ± 0.9	15.7 ± 0.5	14.0 ± 0.9
577	7.4 ± 0.3	6.3 ± 0.2 **	4.6 ± 0.2 ***	5.1 ± 0.2 ***	12.3 ± 0.6	12.7 ± 0.6	10.4 ± 0.8 *	13.0 ± 0.7
599	7.8 ± 0.3	8.3 ± 0.3	8.6 ± 0.3 *	8.5 ± 0.5	12.3 ± 0.9	10.1 ± 0.9	11.0 ± 0.7	12.0 ± 0.6

**Table 4:** Standard deviation, proportion of variance, and cumulative proportion explained by principal components (PC1–PC7) through analysis of PCA from the data of one-year-old and two-year-old *Picea abies* seedlings.

Importance of Components (One-Year-Old)	PC1	PC2	PC3	PC4	PC5	PC6	PC7	PC8
Standard deviation	1.794	1.149	1.047	0.964	0.799	0.622	0.485	0.389
Proportion of Variance	0.403	0.165	0.137	0.117	0.080	0.048	0.0295	0.019
Cumulative Proportion	0.403	0.569	0.706	0.823	0.903	0.951	0.981	1.000
**Importance of Components** **(Two-Year-Old)**	**PC1**	**PC2**	**PC3**	**PC4**	**PC5**	**PC6**	**PC7**	**PC8**
Standard deviation	1.565	1.318	1.084	0.989	0.921	0.632	0.573	0.246
Proportion of Variance	0.307	0.218	0.147	0.122	0.106	0.050	0.041	0.008
Cumulative Proportion	0.307	0.525	0.672	0.795	0.901	0.951	0.992	1.000

**Table 5:** Individual proportion of the traits to the 1st and 2nd components from the data of one-year-old and two-year-old *Picea abies* seedlings.

Trait	One-Year-Old		Two-Year-Old	
	1st Component	2nd Component	1st Component	2nd Component
CAT	0.48201	0.02427	0.61008	0.01850
APX	0.51520	−0.03179	0.45603	0.24341
POX	0.43443	0.18383	0.19429	−0.33059
GR	0.50361	−0.01278	0.60793	0.03309
SOD	0.19564	−0.54432	0.03128	−0.15240
DPPH	0.10827	0.65602	−0.09389	0.59030
ABTS	−0.00743	0.37482	−0.02679	0.67699
HEIGHT	0.09022	−0.31195	−0.04462	−0.00342

**Table 6:** Description of *Picea abies* seeds plantation in Lithuania.

	Plantation Code	Regional Division of State Forestry	Forestry Sector	Geographical Position	Block	Site	Area (ha)	Year of Enrolment
*P. abies*	08ESP031	Trakai	Būda	54°54′38.7″ N 24°18′01.2″ E	216	16, 17	5.32	2002
		Half-sib families: 124, 417, 454, 457, 463, 477, 541, 548, 577, 599						

## Data Availability

Data will be made available on request.

## Supplementary Materials

The following supporting information can be downloaded at: https://www.mdpi.com/article/10.3390/plants13152021/s1, Figure S1: principal component analysis (PCA) displaying the first and second components (PCs) from the data of one-year-old and two-year-old *Picea abies* seedlings in (A) control, (B) CP1, (C) CP2 and (D) EMF2. Figure S2. Principal component analysis (PCA) displaying the first and second components (PCs) from the data of two-year-old *Picea abies* seedlings in (A) control, (B) CP1, (C) CP2 and (D) EMF2.
