# Supplementary material for: Seed Treatment with Cold Plasma and Electromagnetic Field: Changes in Antioxidant Capacity of Seedlings in Different Picea abies (L.) H. Karst Half-Sib Families
Source: Plants (Basel). 2024 Jul 23;13(15):2021. doi: 10.3390/plants13152021 (PMC11314105; doi:10.3390/plants13152021)
Supplement: Supplementary file 1 [file plants-13-02021-s001.zip › plants-3120034-supplementary.pdf]

SUPPLEMENTARY FIGURES

One-year-old

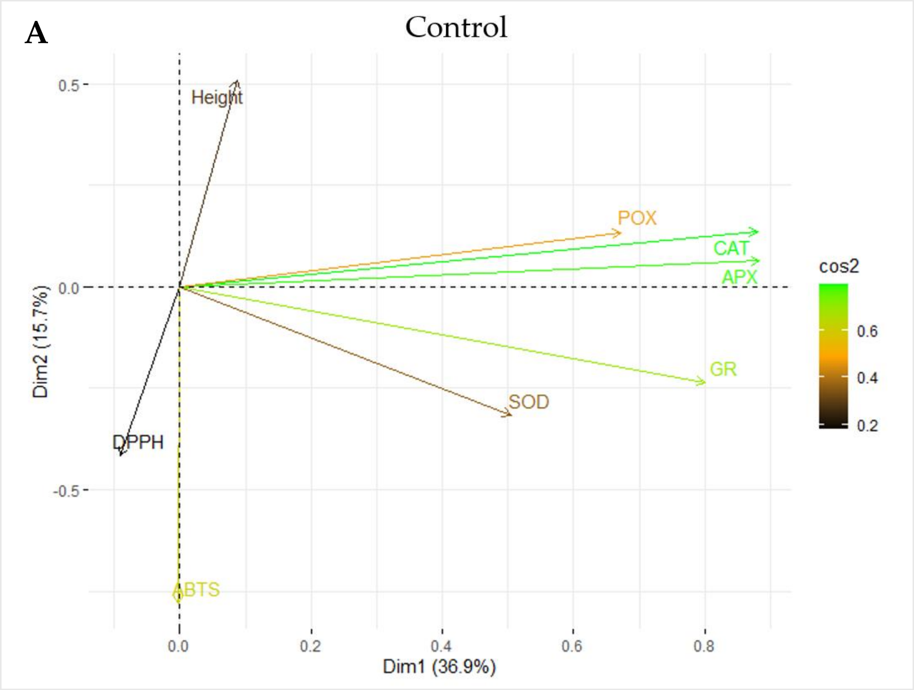

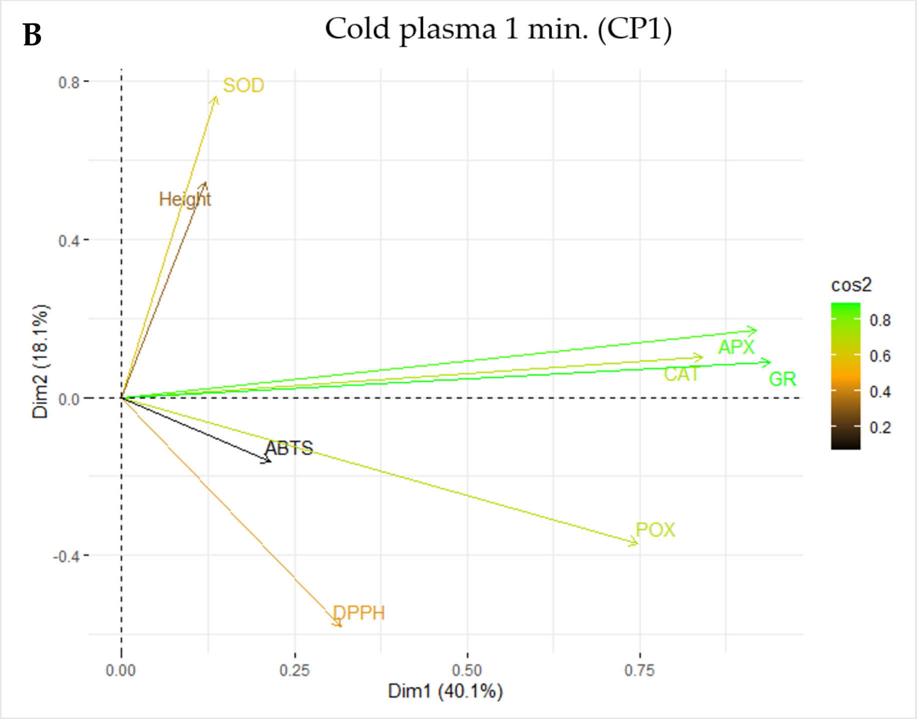

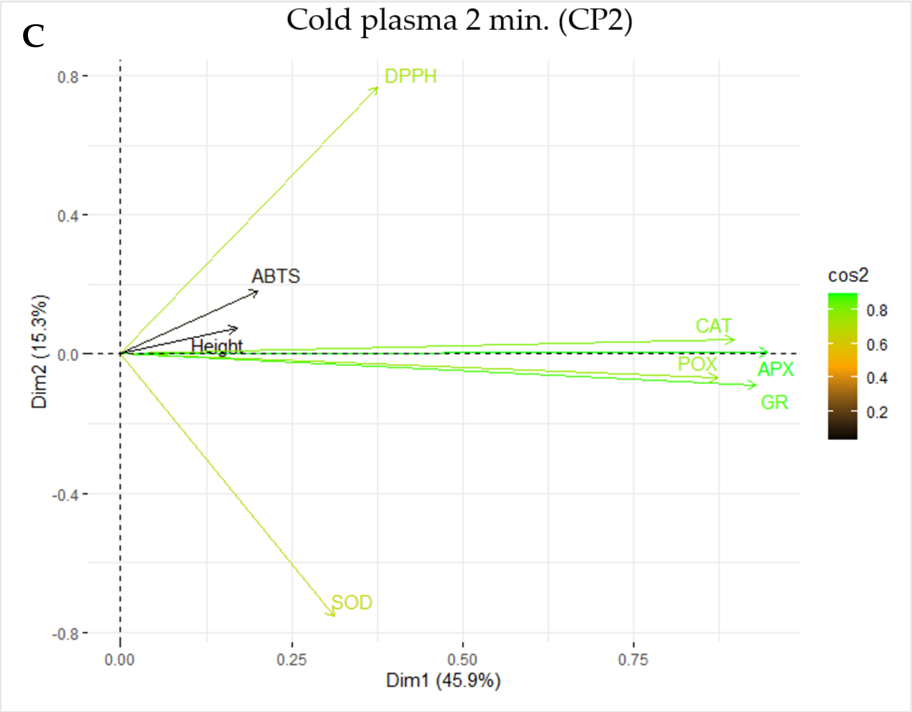

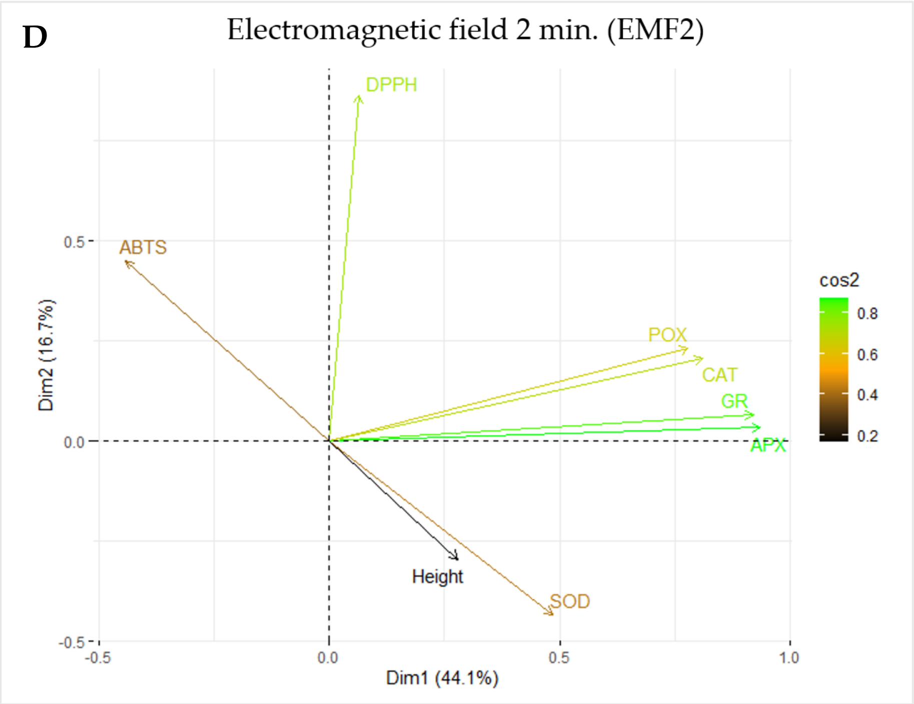

Figure S1. Principal component analysis (PCA) displaying the first and second components (PCs) from the data of one-year-old *Picea abies* seedlings in **(A)** control, **(B)** CP1, **(C)** CP2 and **(D)** EMF2. The loading vectors show the correlation between different variables. Colors of vectors from black to green represent 640 different squared cosine (Cos2) values.

Two-year-old

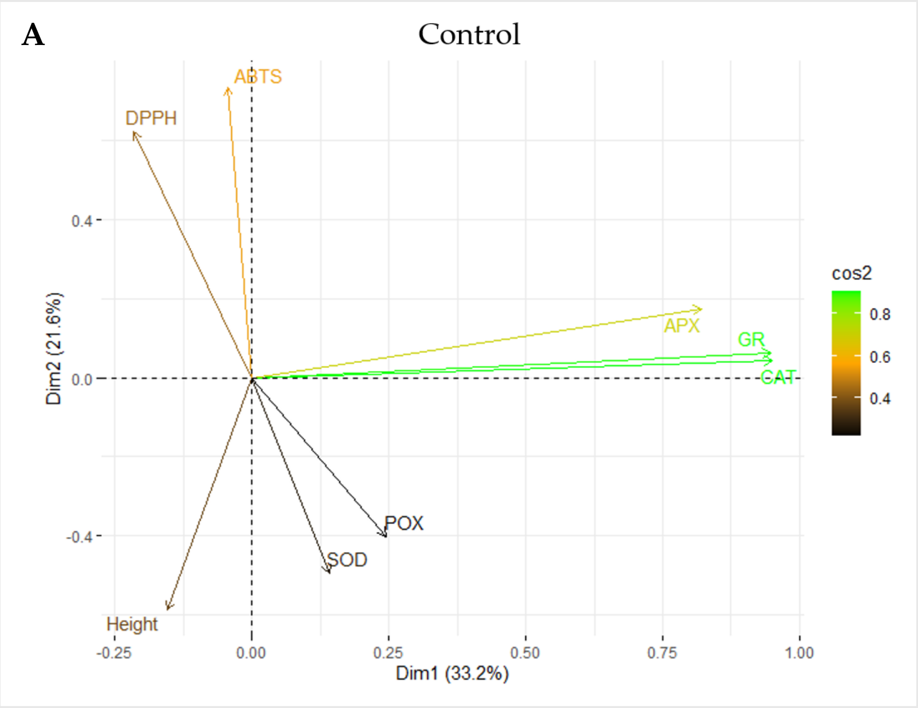
A
Control
ABTS
DPPH
APX
GR
CAT
POX
SOD
Height
cos2
0.8
0.6
0.4
0.4
0.0
-0.4
Dim2 (21.6%)
-0.25
0.00
0.25
0.50
0.75
1.00
Dim1 (33.2%)

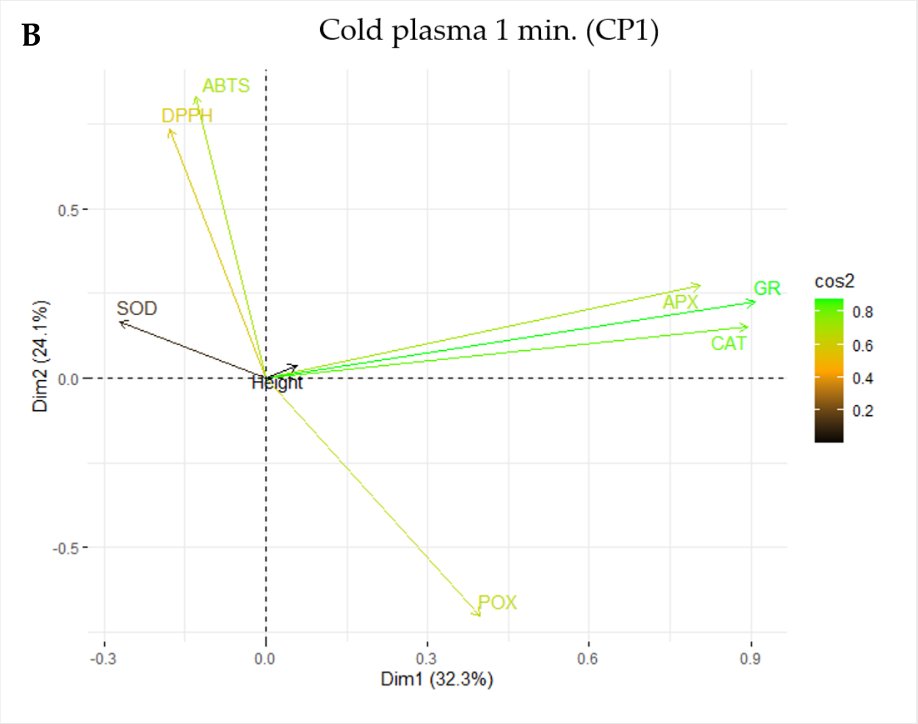
B
Cold plasma 1 min. (CP1)
ABTS
DPPH
SOD
APX
GR
CAT
Height
POX
cos2
0.8
0.6
0.4
0.2
0.5
0.0
-0.5
Dim2 (24.1%)
-0.3
0.0
0.3
0.6
0.9
Dim1 (32.3%)

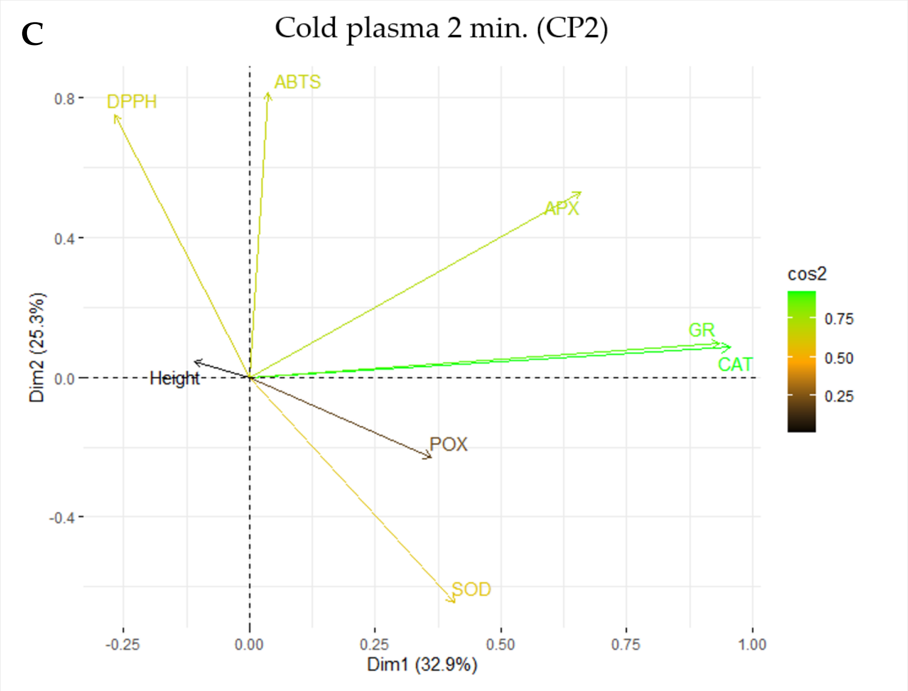

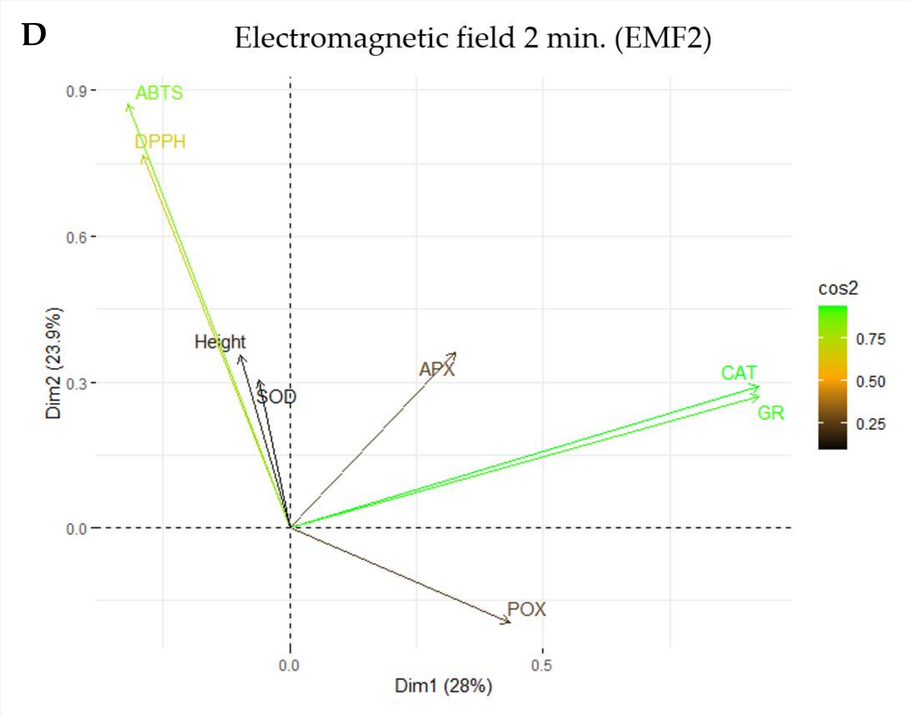

Figure S2. Principal component analysis (PCA) displaying the first and second components (PCs) from the data of two-year-old *Picea abies* seedlings in **(A)** control, **(B)** CP1, **(C)** CP2 and **(D)** EMF2. The loading vectors show the correlation between different variables. Colors of vectors from black to green represent 640 different squared cosine (Cos2) values.
